# Diagnosis of Bone Mineral Density Based on Backscattering Resonance Phenomenon Using Coregistered Functional Laser Photoacoustic and Ultrasonic Probes

**DOI:** 10.3390/s21248243

**Published:** 2021-12-09

**Authors:** Lifeng Yang, Chulin Chen, Zhaojiang Zhang, Xin Wei

**Affiliations:** 1 School of Optoelectronic Science and Engineering, University of Electronic Science and Technology of China, Chengdu 610054, China; charlin@std.uestc.edu.cn (C.C.); z15912188054@gmail.com (Z.Z.); weixin@std.uestc.edu.cn (X.W.); 2 Optoelectronic Imaging and Biophotonics Laboratory, University of Electronic Science and Technology of China, Chengdu 610054, China

**Keywords:** backscattering resonance, photoacoustic signal, osteoporosis diagnosis, fracture risk

## Abstract

Dual-energy X-ray absorptiometry (DXA) machines based on bone mineral density (BMD) represent the gold standard for osteoporosis diagnosis and assessment of fracture risk, but bone strength and toughness are strongly correlated with bone collagen content (CC). Early detection of osteoporosis combined with BMD and CC will provide improved predictability for avoiding fracture risk. The backscattering resonance (BR) phenomenon is present in both ultrasound (US) and photoacoustic (PA) signal transmissions through bone, and the peak frequencies of BR can be changed with BM and CC. This phenomenon can be explained by the formation of standing waves within the pores. Simulations were then conducted for the same bone µCT images and the resulting resonance frequencies were found to match those predicted using the standing wave hypothesis. Experiments were performed on the same bone sample using an 808 nm wavelength laser as the PA source and 3.5 MHz ultrasonic transducer as the US source. The backscattering resonance effect was observed in the transmitted waves. These results verify our hypothesis that the backscattering resonance phenomenon is present in both US and PA signal transmissions and can be explained using the standing waves model, which will provide a suitable method for the early detection of osteoporosis.

## 1. Introduction

Osteoporosis is a metabolic bone disease that worsens as the individual ages. Affected individuals have a high risk of fractures due to increased bone fragility and increased bone porosity. The World Health Organization (WHO) defines osteoporosis as a bone mineral density (BMD) of 2.5 standard deviations (SDs) or less below the young adult mean. However, most osteoporosis cases are undiagnosed until a fracture occurs, which is costly to treat and is associated with high morbidity. X-ray-based BMD measurement represents the gold standard for osteoporosis diagnosis and fracture risk assessment. Dual-energy X-ray absorptiometry (DXA) is widely used to diagnose osteoporosis [1]. It can expose 60% to 80% of the variability in bone strength and assist in the decision making and monitoring of treatment progression. However, other mechanical factors cannot be detected, such as microstructure, collagen, etc., which are also important in determining the fracture risk of bone. The accuracy of DEXA, especially related to fracture risk, has major limitations [2,3]. Twenty-five percent of people with normal bone density also have a fracture risk, but these people cannot be detected with DXA [4], which leads to doubts regarding the accuracy of DXA. DXA, with bone density as the measurement target, is insensitive to changes in bone collagen and bone microstructure. Simultaneously, there are doubts regarding its potential safety because DXA may destroy the cross-linked structure of bone collagen during the measurement process. In recent years, Wang [5] and Carrin [6] confirmed that bone collagen is an important factor affecting bone structure and elastic modulus. French Sophie [7] attempted to use BMD in combination with trabecular bone score (TBS) to improve DXA’s accuracy in osteoporosis detection. Diane et al. [8] found that the combination of TBS and DXA could find 66% to 70% of patients with a fracture risk who were missed by DXA alone. The above cases indicate that it is difficult for DXA to identify patients with early fracture risk, and nearly 30% to 50% of patients are neglected owing to the standard leak point [9]. Some researchers found that the bone density of antlers is very low but that antlers have high toughness [10], and that collagen degradation causes a sharp increase in bone brittleness under the same bone density [11,12]. Willett [13] reported that the cross-linking of collagen is positively correlated with strain toughness, and confirmed that the mechanical properties of bone are related to the characteristics of collagen. New evidence reveals that in addition to BMD, which is a major parameter of bone strength [14,15], other mechanical factors, including micro-architecture, post-yield mechanical properties, and bone collagenous matrix, are also important in determining the fracture risk of bone [9]. The relationship between the organic matrix and the mineral content is important for early fracture prediction, but it cannot be explicitly modeled.

Since osteoporosis is preventable if at-risk populations are detected early, diagnostic techniques are of utmost importance. As osteoporosis is a slowly progressing disease, early detection is critical. Compared with DXA, quantitative ultrasound is a cheap, convenient, and non-radiation-based approach to detecting osteoporosis. Noale [4] investigated 8681 subjects and found that calcaneal Quantitative ultrasound (QUS) was similar to DXA in predicting the risk of fracture caused by osteoporosis. Qin [16] used QUS to detect ultrasound speed and bone machinery and found the correlation between Young’s modulus and bone density, respectively. Janne [17] analyzed the direct relationship between several variables of ultrasound signals in the frequency domain and pointed out that bone intensity and ultrasound parameters has a strong correlation at 3.5 MHz. At present, the main reason why QUS cannot be used to define fracture risk levels is that QUS results require new WHO standards. However, due to the damage of DXA to bone, QUS is often used in the investigation of low-risk osteoporosis and evaluation of fracture treatment results [18]. Traditional techniques measure the BMD as an index of bone porosity, whereas more current technologies analyze the bone’s microstructure [19,20].

Recently, combined backscatter ultrasound (US) and backpropagation (BR) photoacoustic (PA) measurements have enabled advances in assessing bone integrity [21]. Lashkari [22] reported that a combined photoacoustic and ultrasonic dual mode can be used in the early stage. In comparison to PA, US was capable of generating detectable signals from deeper bone sublayers. However, while US signal variations with changes in the cortical layer were insignificant, PA proved to be sensitive even to minor variations of the cortical bone density [23,24]. A problem that needs to be solved from PA/US dual mode in vitro to in vivo testing is how to remove the influence of the skin and soft tissue on the bone surface on the signal. The key goal of this study was to verify that the BR phenomenon is present in both US and PA signal transmissions through bone, and the peak frequencies of BR can change with BMD and CC, which has the potential to be used to determine which part is from the bone tissue instead of the collagen of the skin in early detection.

## 2. Materials and Methods

### 2.1. Experimental Setup

**Figure 1:**
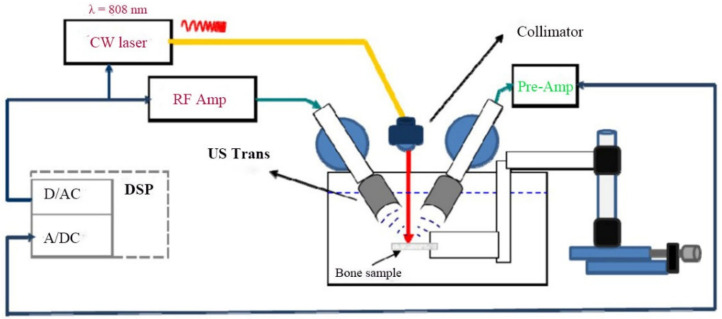
Schematic of the experimental setup.

The experiment was set up is shown in Figure 1. It allowed for US and PA tests to be conducted on the same sampling point on the bone. The PA excitation was generated by a CW 808 nm diode laser (Jenopotik AG, Jena, Germany), and the laser intensity was modulated by a software function. The laser fluence on the target surface was about 40 mJ/cm^2^ for 5 ms per measurement, which is far below the ANSI safety standard of 1425 mJ/cm^2^ for 808 nm laser exposure of the skin. A collimator was used to ensure parallel rays that would spread minimally when propagating toward the sample. The collimated laser beam was 2 mm in diameter on the sample. For generating US waves, a 3.5 MHz focused ultrasonic transducer was used (V382, Olympus NDT Inc., Waltham, MA, USA). The backscattered waves were then detected by a 2.25 MHz focused transducer (V305, Olympus NDT Inc., Waltham, MA, USA). Both transducers and the bone sample were submerged under water for acoustic coupling. The laser beam was set perpendicular to the sample, and its point of incidence was used to adjust the focal point of both transducers to the same point on the sample. The angle between the laser beam and each transducer’s center axis was 27° according to a method previously used [22].

### 2.2. Bone Specimens

**Figure 2:**
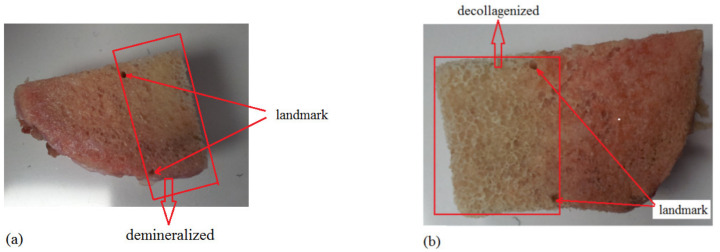

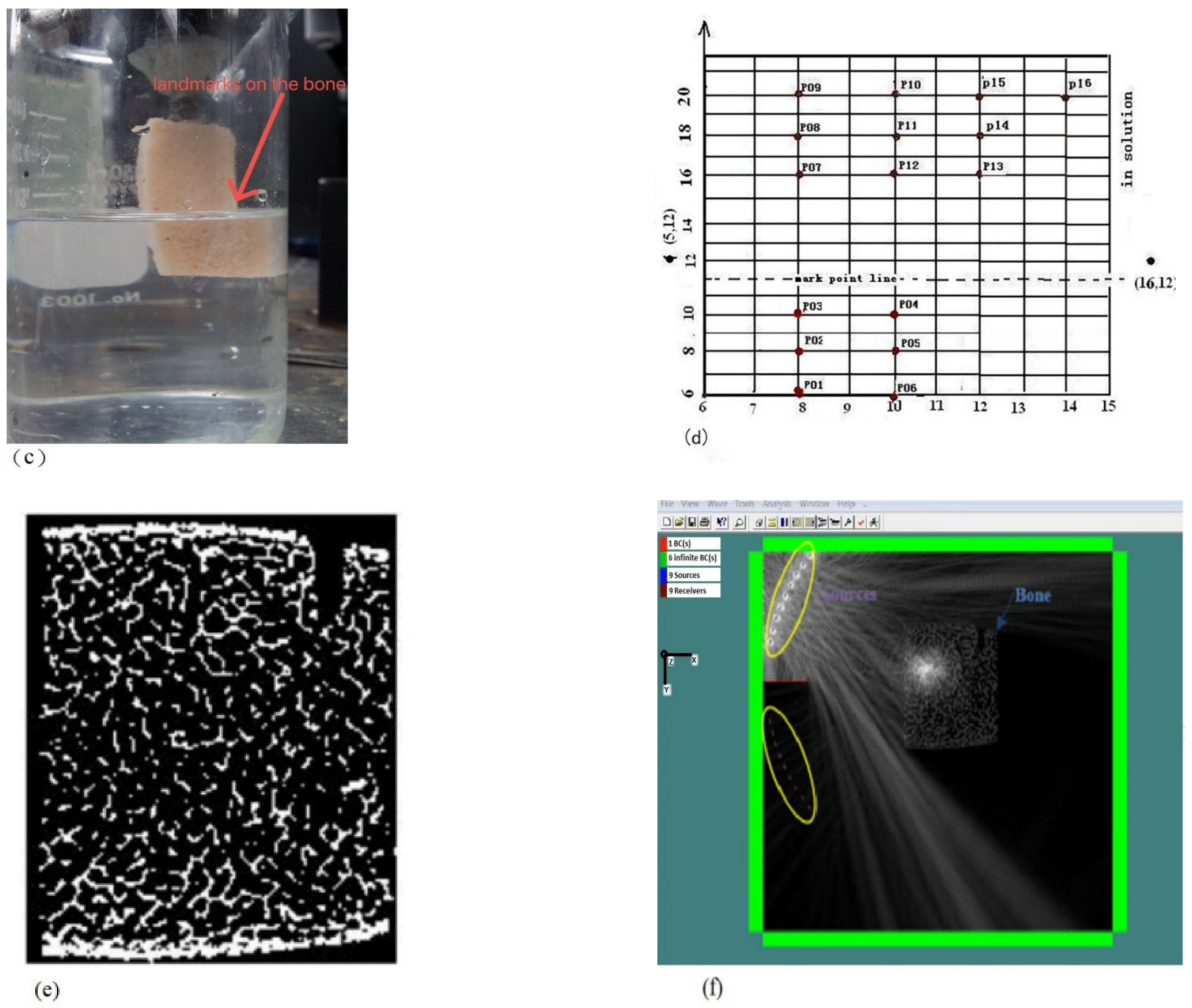
(**a**) Landmarks (two points indicated on the sample) were artificially created to distinguish the measurement points and mark the horizontal line below which the sample was immersed in the solution agent. (**b**) Landmarks (two points indicated on the sample) were artificially marked on the decollagenized sample. (**c**) The indicated line coincides with the surface of the solution. (**d**) The relative position of the 16 measured points landmarked on one sample. (**e**) DICOM image of a bone sample scanned using µCT; the extra void spaces were cropped out. (**f**) Simulations were conducted using a commercial standalone software package for computational ultrasonics (Wave3000^®^).

Bone samples were harvested from the femur and ischium of the same cattle and cut into six rectangular-like samples of similar size. The samples were stored in a refrigerator before processing or measurement, and thermally equilibrated at room temperature before the experiment or measurement. These specimens were randomly divided into two groups and treated with different agents to reduce their mineral or collagen contents [25,26]. Landmarks (two mark points on the sample) were artificially created to distinguish the measurement points before treatment, and then the three samples were treated with 50% ethylenediaminetetraacetic acid (EDTA) buffer solution (pH = 7.7) on the same side of the sample to demineralize the bones, simulating osteoporosis, as shown in Figure 2a. The other three samples were treated with a 5% liquid sodium hypochlorite solution to reduce the CC, as shown in Figure 2b. Landmarks indicated the position of the immersion solution, as shown in Figure 2c, and each measuring point was used as the coordinates (relative position) to indicate the specific position on the sample, as shown in Figure 2d. After preparation, these samples were immersed in phosphate-buffered saline, stored in a refrigerator (−20 °C), and thawed before measurements. The signal of each specimen was detected at the same point before and after sample treatments. The treatment procedures of demineralization and decollagenization were described in detail in the literature [25,26].

### 2.3. µCT Scanning

On the basis of magnetic resonance imaging (MRI), FE-based estimation of skeletal mechanical capacity involves a number of image-processing and calculation steps [27,28,29]. The bone sample was first analyzed using micro-computed tomography (µCT) scanning where cross-sectional slices of bone were captured as Digital Imaging and Communications in Medicine (DICOM)images. Each slice was 15 µm thick, and the entire sample yielded 300 images.

The inter distance between the trabeculae of a bone sample along the axis of the source wave was measured by analyzing µCT images using MATLAB (R2020a). The images were then used in Wave3000 (CyberLogic, inc., 2020), which is numerical software for simulating acoustic wave propagation through trabecular bone samples in the defined media and boundaries. This helped to simulate the propagation of ultrasound waves in trabecular bone structure, thereby providing a better understanding of the coherent backscattering effect, enabling analysis of the relationship between resonance frequencies and trabecular bone microstructure [30]. Trabecular bone is considered to consist of two materials: (1) trabeculae: cortical bone (density 1.85 g·cm^−3^, bulk velocity 2900 m·s^−1^, and shear velocity 1300 m·s^−1^); (2) inner and outer coupling medium: water (25 °C, density 1.00 g·cm^−3^, bulk velocity 1497 m·s^−1^, and shear velocity 3.54 m·s^−1^). The values of the acoustic properties for cortical bone and water were obtained from the Wave 3000^®^ material library. Simulation geometry was matched with the experimental measurement geometry. The distance between the transducers was 10 cm and the diameter of the transducers was 2.54 cm. The acoustic source was configured to be identical with the actual transducers (center frequency of 2.25 MHz) used in the experimental measurements [31]. The results of the simulation were then verified experimentally. The purpose of the experiment was to show that the resonance phenomenon [32] is present in bone US and PA signals, thereby justifying the simulation efforts. Further simulations in Wave3000^®^ were conducted by artificially enlarging the µCT images to examine the relationship between peaks in the resonance frequency spectrum and the inter-trabecular distance spectrum, as shown in Figure 2e,f.

### 2.4. Photoacoustic Backscattering

PA refers to the emission of sound waves from a material after absorbing light waves. Studies showed that a coherent backscattering effect similar to that of US in trabeculae bone can be detected as frequencies higher than 1MHz [25]. Due to the complex nature of bone media, many parameters have been defined for the prediction of the backscattering coefficient (BSC) in the trabeculae. The BSC is a measure of the attenuation of signal caused by scattering at angles from 90° to 180°. Coherent backscattering occurs when the waves propagate through a medium with scattering points of size comparable to the wavelength. This creates the effect termed coherent backscattering because it usually creates a sharp peak in the amplitude vs. frequency graph of the reflected wave in the direction of the backscatter. The frequency of the source wave that generates this coherent backscattering is called the resonance frequency. Some phenomena can explain this sharp peak where standing waves are formed between the inter-trabeculae walls when these waves are scattered multiple times within the trabeculae.

For the formation of standing waves, the resonance frequency can be predicted using the equation for finding standing wave harmonic frequencies:

(1) $$f_{n}=n(\frac{v}{2l})$$

Where *f_n_* is the harmonic frequency, n is the harmonic number, v is the speed of sound in a medium, and l is the length between the nodes, which is the inter-trabecular length.

Equation (1) was used to find the expected resonance frequencies, which were then compared to the resonance frequencies of the simulations and experiments.

### 2.5. Quantitative Ultrasound (QUS) and Photoacoustic (PA) Measurements

An artificial horizontal landmark line in each sample was used to distinguish the measurement points. The points below this landmark of the sample were immersed in the liquid solution. The points above this landmark were not affected by the solution (Figure 2a,b), so were not demineralized or decollagenized. The points above the landmark were used as a reference to reveal the changes in the US/PA signal due to factors before and after demineralization/decollagenization. The frequency spectra of PA and US signals were normalized by those spectra. In this study, we used the apparent integrated backscatter/back-propagating (AIB) [17,23,31] parameter, which is determined by frequency averaging (integrating) the ratio of the power spectrum of the signal (Pb) over the power spectrum of a reference signal (Pr) over the chirp frequency range:

(2) $$\mathrm{US}\;\mathrm{or}\;\mathrm{PA}\;\mathrm{AIB}=\frac{1}{\Delta f}\int_{\Delta f}10\log_{10}\left(\frac{P_{b}\left(f\right)}{P_{r}\left(f\right)}\right)df$$

The apparent US or PA integrated backpropagating signal was calculated using Equation (2), where Pb is the power spectrum of the signal and Pr is the power spectrum of a reference US or PA signal. To eliminate the transfer function effect of the transducer and other instruments, the spectra of the US and PA signals were normalized with their respective reference spectra.

## 3. Results

### 3.1. BoneSamples and µCT Scanning

**Figure 3:**
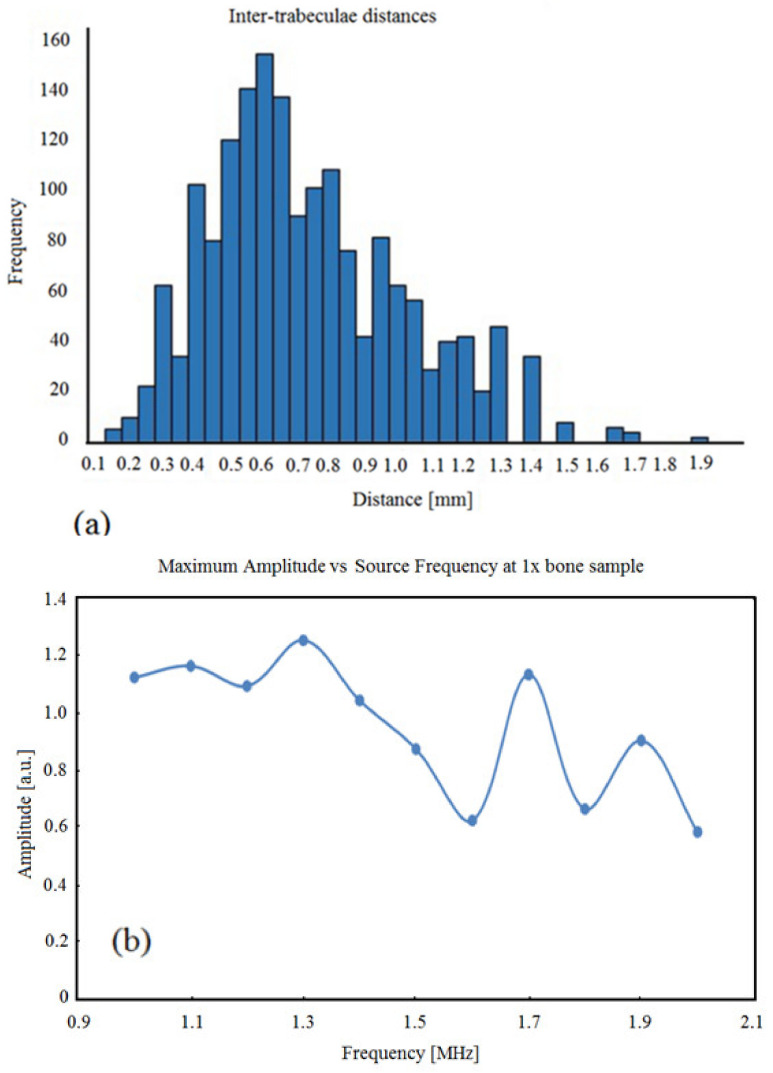
(**a**) Histogram of inter-trabeculae distances (mm) in the 25 slices of bone used for simulation; (**b**) maximum amplitude vs. source frequency for the original bone sample.

The consolidated inter-trabeculae distances of the 25 slices of bone used in the simulations are shown in Figure 3a. The shape of the distribution is similar to that of a skewed Gaussian distribution centered around 0.6 mm, which was the most frequent value. Note that since this is a histogram, 0.6 mm is the bin value and not the actual value of the inter-trabeculae. The actual values would fall in the range 0.56–0.65 mm. Using 0.6 mm as the value for length in Equation (1) and using 1540 m/s as the speed of 3.5 MHz US in water, the theoretical expected resonance frequencies are f1 = 1.28 MHz and f2 = 2.56 MHz. Only the first two harmonics were considered, since the amplitude decreases exponentially as the harmonic number increases, as shown in Figure 3b.

### 3.2. Simulation Results

The simulation was completed according to the setup with 1× inter-trabeculae distance bone samples. For each run of the simulation, i.e., for each frequency, the maximum amplitude from each of the nine receivers was identified and averaged across the receivers. The averaged maximum was plotted against the frequency of the source US waves, as shown in Figure 3b.

The original bone sample size showed three peak frequencies, located at 1.3, 1.7, and 1.9 MHz. The first peak at 1.3 MHz is close to the expected resonance frequency of 1.28 MHz calculated previously. If the resonance frequency values of 1.7 and 1.9 MHz are used in Equation (1), the inter-trabeculae distances that could have generated these resonances would be 0.45 and 0.4 mm in length, respectively. This showed that a small peak occurred in the bin value of 0.4 mm, as shown in the histogram in Figure 3b. This could explain why 1.7 and 1.9MHz are also resonance frequencies in this simulation.

### 3.3. Photoacoustic and Ultrasound AIB Comparison

**Figure 4:**
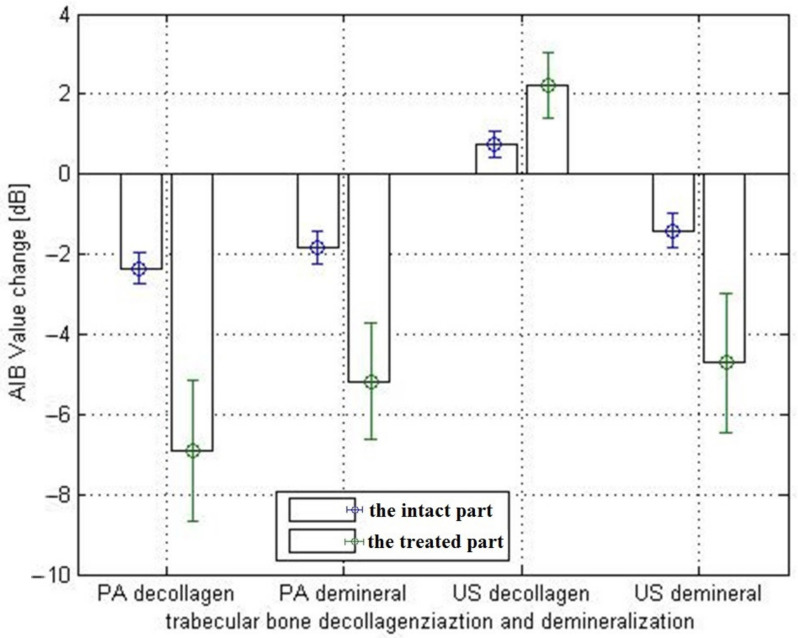
PA and US AIB value variation histogram before and aftertreatment for intact and treated parts of the bone samples.

Figure 4 shows that both PA and US AIB values significantly reduced in the demineralized part of the bone.PA signals decreased significantly in the decollagenized part of the bone, whereas US parameters increased slightly in the decollagenized part. Several points of each sample (demineralized or decollagenized) were tested, and the average changes in the US and PA AIB values of each group of samples are shown in Table 1. The averaged correlation coefficients are weak to moderate for the intact parts of all samples with r values ranging from 0.468 to 0.632, which are relatively small compared with those between the US AIB and μCT. Additionally, the PA AIB showed weak correlation with μCT before and after either treatment, as previously reported [22,31].

### 3.4. Actual Experiment Results

**Figure 5:**
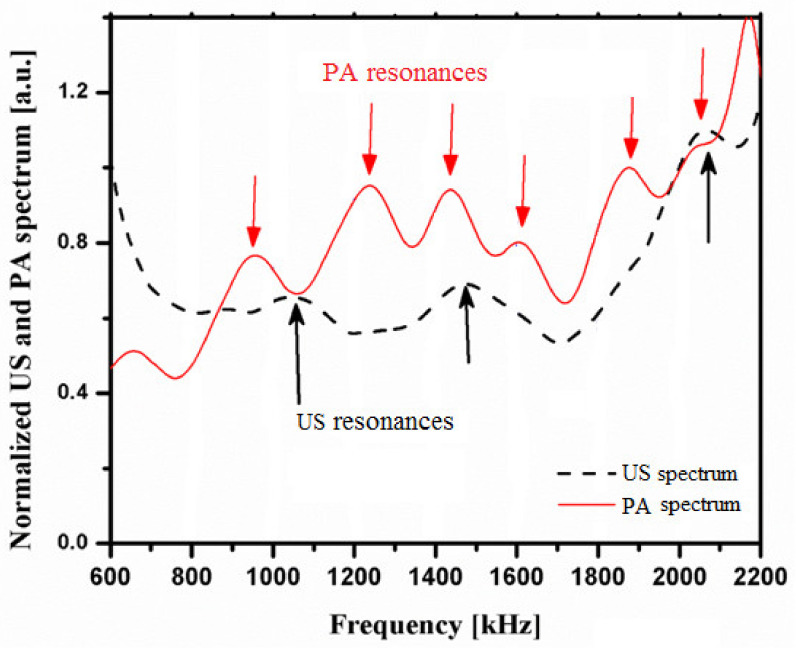
Normalized spectra for US and PA signals vs. source frequency with 1× inter-trabeculae distance bone samples.

Compared with the PA spectrum in the simulation, the PA peak frequencies from the experimental results differed from the peaks in the simulation results both in position and in number. There are only three peaks, at 1.3, 1.7, and 1.9 MHz, in Figure 3b, whereas the PA spectra in Figure 5 have six peaks. This seems to justify the findings of a previous study that showed PA to be more sensitive than US to bone characteristics [31]. Notably, even though the simulated resonance frequency values match the theoretical values used in both simulations, the shape of the frequency spectra differed considerably between the two simulations.

Comparing the US spectrum in Figure 5 to that in Figure 3b, a shift in the spectrum can be observed. The three peaks in the simulation results occurred at 1.3, 1.7, and 1.9 MHz, whereas the peaks in the experimental results occurred at 1.1, 1.5, and 2.1 MHz. The differences for all three peaks are ± 0.2 MHz. Arrows in Figure 3b and Figure 5 refer to the positions of the peaks.

**Figure 6:**
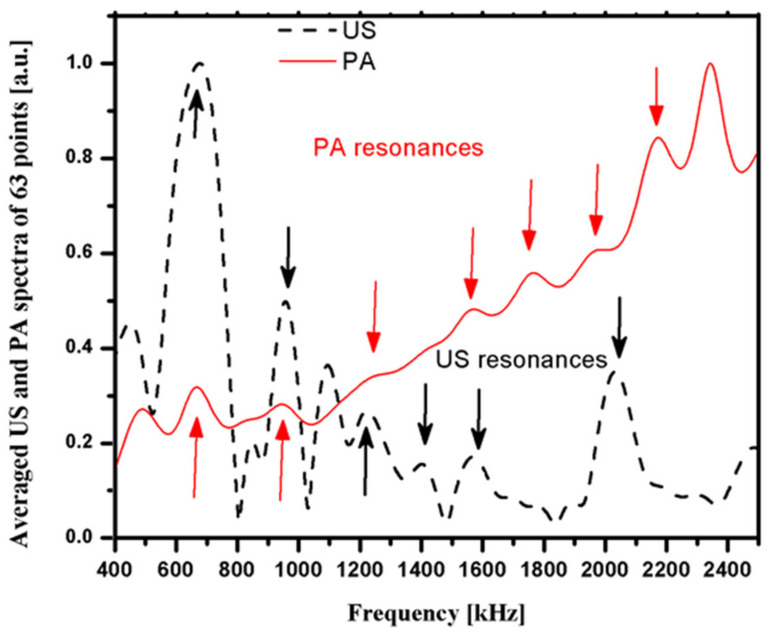
Normalized spectra for US and PA signals vs. source frequency with 63 measurement points.

By increasing the number of measurement points to 63 on the same sample, we normalized the average of the 63 detected signals to generate the US and PA spectra in Figure 6, where some similar resonances on both PA and US can be observed.

In Figure 5 and Figure 6, several peaks occur in both the US and PA spectra. This shows that the resonance phenomenon is present in both PA and US signals.

**Figure 7:**
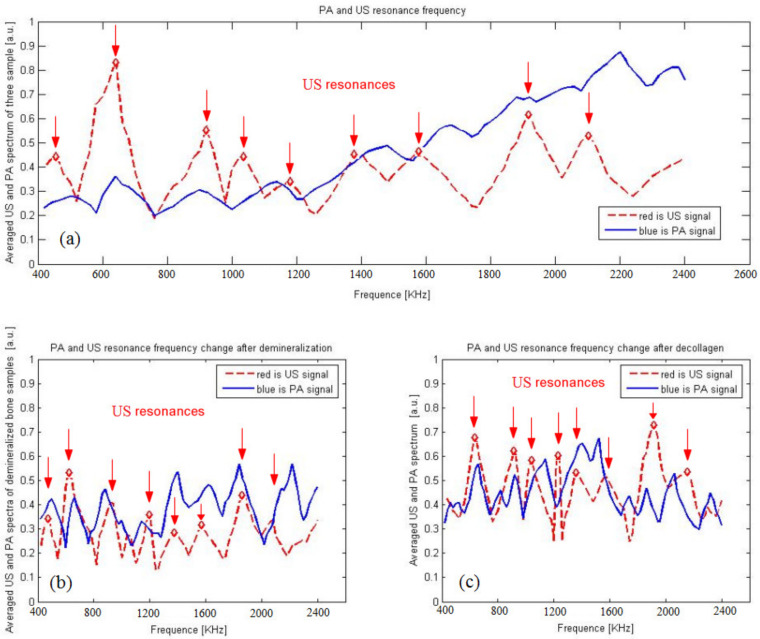
Normalized spectra for US and PA signals vs. source frequency. (**a**) PA and US resonance frequency. (**b**) PA and US resonance frequency change after deminieralization. (**c**) PA and US resonance frequency change after decollagenization.

Another experiment was conducted using the treated and intact parts of the same samples, and the results are shown in Figure 7. There are several peaks in both the US and PA spectra in Figure 7a. We found that the PA has a resonance peak similar to that of the ultrasound resonance after demineralization and decollagenization; there is a little difference in the resonance peaks. Conversely, the resonance peaks after demineralization show obvious changes in both the PA and US spectra in Figure 7b, and the resonance peaks after decollagenization have obvious changes in the PA spectra in Figure 7c, indicating that the PA resonance peaks have better sensitivity, especially to changes in collagen. There are important underlying laws that need to be further studied. This seems to justify previous studies that showed PA to be more sensitive to bone characteristics than US.

## 4. Discussion

Local resonance characteristics occur in PA and US in cancellous bone. The laser is not only absorbed by the collagen in the bone tissue cells, but also by the inorganic calcium bone tissue. The light energy is absorbed by different bone tissues and converted into ultrasonic waves that propagate in the multilayer bone structure.

When sound waves propagate in a composite material with a periodic or quasi-periodic structure, due to the large gap between the acoustic characteristics of two or more materials forming the composite material, the phenomenon of local resonance of the acoustic signal occurs in the deep layer.

This study shows that the PA spectrum exhibits more resonance frequencies or constructive interferences, and there are similar resonance frequencies in PA and US spectra (Figure 5 and Figure 6). PA is not only sensitive to mineral density variation but also to collagen content. On the other hand, US backscattering is mainly sensitive to BMD variation (Figure 7).

Since simulations are based on solving a single viscoelastic wave equation given some fixed material properties, the aim is to find a solution in an idealized situation where everything happens according to the equation. However, because the trabeculae is such a complex structure, in actual experiments, the US waves may interact in a different manner than dictated by the equation. Variations that could not be accounted for in the simulation, such as inhomogeneous material properties due to varying amounts of mineralization, could be why the experimental results do not match those of the simulation, which needs a better bone mathematical model based on µCT or MRI data to judge the influence of collagen on the signal.

The PA and US dual-mode osteoporosis detection method not only detects the changes in bone density, but also the characteristics of osteoporosis such as the loss of collagen and microstructure trauma, which will provide a suitable method for detecting the early stages of osteoporosis. However, a large amount of collagen exists on the surface of the skin. A problem that needs to be solved from PA/US dual mode in vitro to in vivo testing is how to remove the influence of the skin soft tissue on the bone surface on the signal. If PA and US signals are to detect the information from the collagen in the bone tissue, it is necessary to exclude the influence of the collagen on the skin surface. Local resonance is formed in cancellous bone tissue, but this phenomenon does not occur in surface skin, fat, and other tissues. This article reports, for the first time, the BR phenomenon in both US and PA signal transmissions through the bone, which will provide a more sensitive method for real-time bone diagnostic techniques in vivo.

## 5. Conclusions

The results from the preceding simulations showed a close correlation between the US resonance frequencies and the first harmonic frequencies of standing waves formed in the most frequently occurring inter-trabeculae distances. The results from the experiments showed the presence of the BR phenomenon in both US and PA signal transmissions through the bone. These results validated our hypothesis that the resonance effect can be observed in both US and PA signal transmissions through the bone, which could be explained by the formation of standing waves. A comparison of the simulation results to experimental results however showed a ±0.2 MHz difference in the resonance frequencies. Therefore, the simulation was not an accurate predictor of the actual experiment. We were able to correctly predict the number of resonance peaks present but the positions of these peaks were inaccurate. Further studies should be conducted in the future to identify more accurate simulation models and to ensure that the resonance effect is dependent only on bone microstructure.

These results verify our hypothesis that the resonance effect can detect the characteristics of osteoporosis, such as the loss of collagen and microstructure trauma, which will provide a suitable method for the early detection of osteoporosis.

## Figures and Tables

**Table 1:** The correlation coefficient between US or PA AIB and microcomputed tomography (μCT) in the treated and intact parts of the samples.

Samples		Intact Part		Treated Part	
		US/μCT	PA/μCT	US/μCT	PA/μCT
Demineralized	1#	0.632	0.327	0.375	0.052
	2#	0.579	0.213	0.252	−0.323
	3#	0.536	−0.107	0.113	−0.357
Average		0.582	0.144	0.247	−0.209
Decollagenized	1#	0.511	0.233	0.394	−0.078
	2#	0.529	0.324	0.287	−0.116
	3#	0.468	0.156	−0.132	−0.096
Average		0.503	0.238	0.183	−0.097

## Data Availability

The data that support the plots within this paper are available from the corresponding author on request basis.
